# Recurrent mutations in a *SERPINC1* hotspot associate with venous thrombosis without apparent antithrombin deficiency

**DOI:** 10.18632/oncotarget.21365

**Published:** 2017-09-28

**Authors:** Wei Zeng, Bei Hu, Liang Tang, Yan-Yan You, Mara Toderici, Maria Eugenia de la Morena-Barrio, Javier Corral, Yu Hu

**Affiliations:** ^1^ Institute of Hematology, Union Hospital, Tongji Medical College, Huazhong University of Science and Technology, Wuhan, Hubei, China; ^2^ Department of Radiation and Medical Oncology, Zhongnan Hospital of Wuhan University, Wuhan, Hubei, China; ^3^ Department of Anesthesiology, Tongji Hospital, Tongji Medical College, Huazhong University of Science and Technology, Wuhan, China; ^4^ Centro Regional de Hemodonaciόn, Universidad de Murcia, IMIB, CIBERER, Murcia, Spain

**Keywords:** venous thromboembolism, antithrombin deficiency, *SERPINC1*, thrombophilia, mutation

## Abstract

Despite the essential anticoagulant function of antithrombin and the high risk of thrombosis associated with its deficiency, the prevalence of antithrombin deficiency among patients with venous thromboembolism (VTE) is very low. However, increasing evidence suggests that antithrombin deficiency may be underestimated. The analysis of *SERPINC1*, the gene encoding antithrombin, in 1,304 consecutive Chinese VTE patients and 1,334 healthy controls revealed a hotspot involving residues 294 and 295 that severely increases the risk of VTE. We detected the c.883G>A (p.Val295Met) (rs201381904) mutation in 11 patients and just one control (OR = 13.6; 95% CI: 1.7-107.1); c.881G>T (p.Arg294Leu) (rs587776397) in six patients but no controls; and c.880C>T (p.Arg294Cys) (rs747142328) in two patients but no controls. In addition, c.881G>A (p.Arg294His) (rs587776397) was identified in one control. These mutations were absent in a Caucasian cohort. Carriers of these mutations had normal antithrombin levels and anticoagulant activity, consistent with results obtained in a recombinant model. However, mutation carriers had a significantly increased endogenous thrombin potential. Our results suggest the existence in the Chinese population of a hotspot in *SERPINC1* that significantly increases the risk of VTE by impairing the anticoagulant capacity of the hemostatic system. This effect is not revealed by current antigen or *in vitro* functional antithrombin assays.

## INTRODUCTION

Venous thromboembolism (VTE), mainly including deep-vein thrombosis (DVT) and pulmonary embolism (PE), is a severe and complex disorder. With an estimated annual incidence rate of 0.13-0.57 per 1,000 individuals in Asians [1–3] and 1.04-1.83 per 1,000 individuals in Caucasians [4–7], VTE is the third leading cause of cardiovascular mortality after coronary artery disease and stroke, and a major global burden of disease [8]. Owing to the extension of life expectancy and population aging, the incidence of VTE in Asian countries has shown a steady increase [8]. Indeed, recent studies indicated that the rate of VTE after major surgery and in hospitalized medical patients is approaching that of Western populations [9]. Thus, persisting in studying the pathogenesis of VTE, which facilitates the identification and prophylaxis of patients at risk, will benefit human health.

VTE is a multifactorial disease. The interaction of genetic and environmental factors and life events plays a dominant role in the pathogenesis of this complex disease [10–12]. Using modern methodologies in genetic epidemiology, studies of twins and families have indicated that VTE is highly heritable, with an estimated heritability of approximately 0.50 and a familial standardized incidence ratio of around 2.50 [13–15]. Unfortunately, few genetic factors that significantly increase the risk of VTE have been identified [16]. Antithrombin deficiency was the first and is so far the strongest thrombophilic factor [16]. This is explained by the key inhibitory action that this anticoagulant serpin exerts on thrombin and other procoagulant proteases [17]. Patients with antithrombin deficiency have a 5-50-fold increased risk of thromboembolism, predominantly in the venous circulation [18, 19]. However, the incidence of antithrombin deficiency among patients with venous thrombosis is low, 1.9-3.5% in Caucasians and 4.1-8.1% in Chinese [20–25]. However, recent evidence suggests that antithrombin deficiency may be underestimated. Indeed, current functional methods to diagnose antithrombin deficiency fail to detect pathogenic mutations in *SERPINC1*, the gene encoding antithrombin [26], and certain mutations in *SERPINC1* may only show pathogenicity under specific conditions [27, 28].

The aim of our study was to validate the potential thrombotic role of c.883G>A (p.Val295Met) mutation of *SERPINC1* found in two patients upon sequencing this gene in 190 Chinese VTE patients with normal antithrombin levels [29]. Here, we report the identification of a recurrent hotspot in *SERPINC1* that severely increases the risk of venous thrombosis in the Chinese population.

## RESULTS

### Baseline characteristics of the subjects

A total of 1304 VTE patients and 1344 healthy controls were enrolled in the current study. The demographic characteristics and genetic data of the study population were previously summarized in detail [30]. Briefly, the case and control groups had similar median ages (52 years), and included a slight predominance of women (51%). Malignancy and lupus anticoagulant were more prevalent in patients than controls (6.2% vs. 0.4% and 5% vs. 0.9% in cases and controls, respectively). Also, a higher incidence of cigarette smoking, immobility, oral contraceptives use, and hormone-replacement therapy was recorded in patients with VTE. In addition, three common polymorphisms, two in the protein C gene (*PROC* c.565C>T, *PROC* c.574_576del), and one in the thrombomodulin gene (*THBD* c.-151G>T), were more prevalent in patients than in controls.

### *SERPINC1* mutation identification

**Figure 1:**
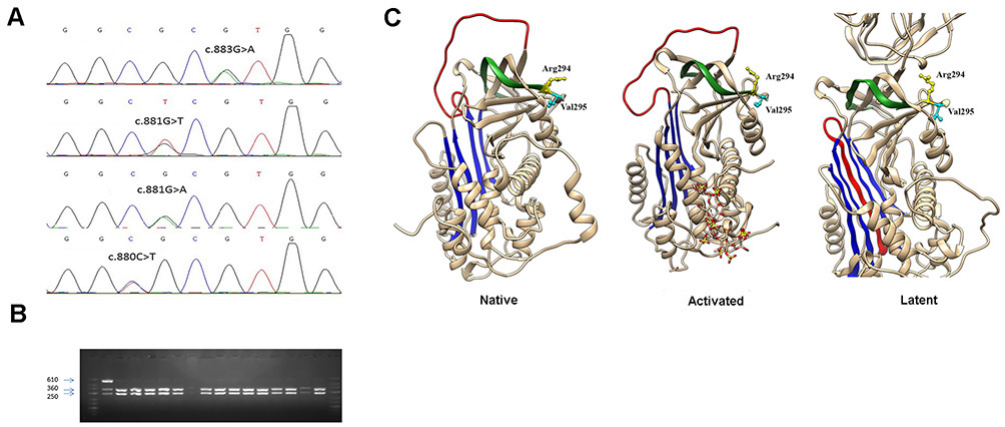
Identification of a mutational hotspot in *SERPINC1* **(A)** Electropherogram of samples carrying all mutations affecting antithrombin Arg294 and Val295 residues. **(B)** Restriction fragments generated with BstUI on the PCR product of *SERPINC1* exon 5 using the primers described in Materials and Methods. **(C)** Localization in the structure of antithrombin of affected residues in the native, activated, and latent configurations. The central A sheet is colored in blue, the reactive center loop in red, and s1B in green. Model building was performed by using SWISS-MODEL and the Swiss-PdbViewer programs [Guex N, Peitsch MC. SWISS-MODEL and the Swiss-PdbViewer: an environment for comparative protein modelling. Electrophoresis 1997; 18: 2714–23] (http://www.expasy.ch/spdbv).

According to the results of restriction analysis done in the present case-control study, 19 patients with VTE and 2 healthy controls were found to carry an allele which could not be digested by BstUI, all in heterozygous state. Surprisingly, when these 21 positive samples were sequenced to validate the result, four different missense mutations were identified: c.883G>A (p.Val295Met) (rs201381904), detected in 11 VTE patients and 1 control; c.881G>T (p.Arg294Leu) (rs587776397), identified in 6 patients; c.880C>T (p.Arg294Cys) (rs747142328), found in 2 patients; and c.881G>A (p.Arg294His) (rs587776397), found in one healthy control (Figure 1). As expected, all these mutations disturbed the sequence recognized by BstUI, explaining why they all render the same restriction pattern. No additional prothrombotic polymorphisms (*PROC* c.565C>T, *PROC* c.574_576del or *THBD* c.-151G>T) were detected in any of the carriers of these *SERPINC1* mutations.

Globally, any mutation affecting this hotspot increased the risk of thrombosis 9.8-fold (95% CI: 2.3-42.4). The most recurrent mutation, c.883G>A, was strongly associated with increased risk of thrombosis (OR = 11.3; 95% CI: 1.5-88.0; P = 0.003). The statistical power for the study was approximately 0.855, with a significance level of 0.05.

Multivariate analysis, including age, gender, smoking, malignancy, lupus anticoagulant, immobility, and oral contraceptives/hormone-replacement therapy confirmed that all these *SERPINC1* mutations significantly increased the risk of VTE (OR = 11.4; 95% CI: 2.6-49.4; P = 0.001), particularly the recurrent *SERPINC1* c.883G>A mutation mentioned above (OR = 13.6; 95% CI: 1.7-107.1; P = 0.013).

**Figure 2:**
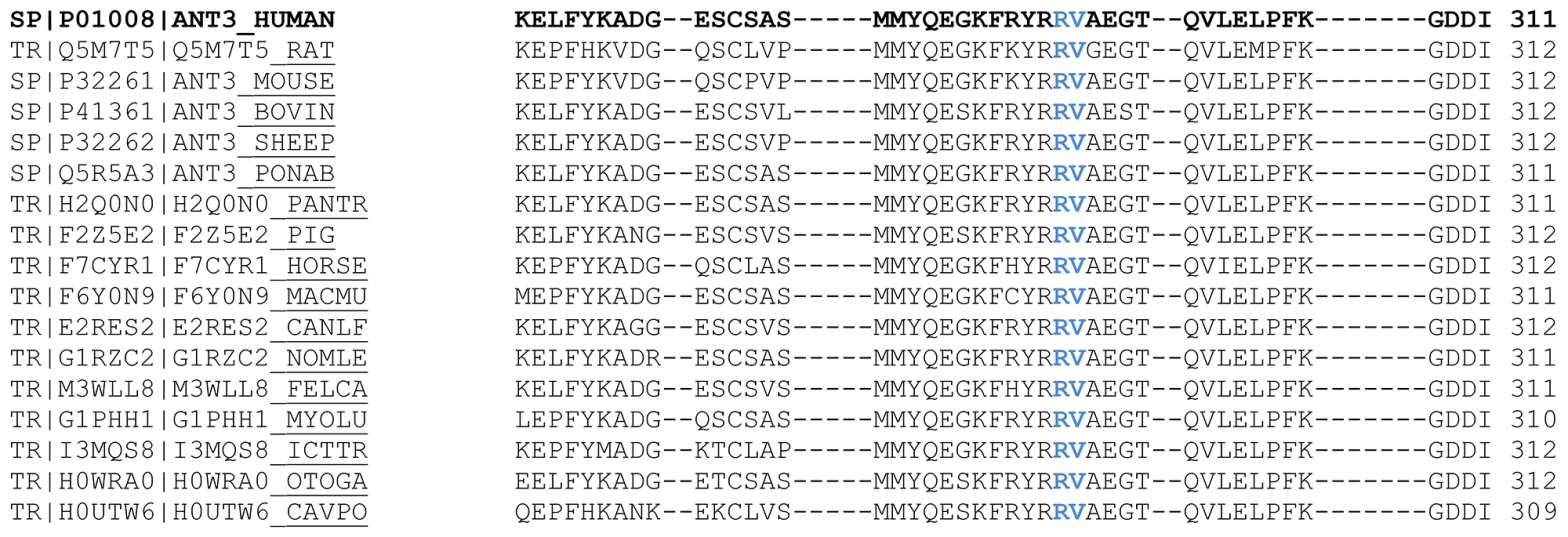
Conservation of Arg294 and Val295 among antithrombins of 17 species Alignments were performed using the Clustal Omega program. Data available at Uniprot (www.uniprot.org/uniprot/)

The hotspot identified affects two residues strongly conserved among antithrombins from different species (Figure 2), but not among the serpin superfamily [31], located at the end of strand 1B (s1B) (Figure 1). None of these mutations had been previously described in patients with antithrombin deficiency (http://www.hgmd.cf.ac.uk/ac/gene.php?gene=SERPINC1).

### Plasma antithrombin levels and anticoagulant potential

Antithrombin antigen level, and two tests evaluating the anticoagulant activity of plasma antithrombin, i.e. anti-FXa and anti-FIIa assays, were done in 20 subjects carrying these four *SERPINC1* mutations and in 30 healthy donors. Mutation carriers had normal antigen levels and anti-FXa activity, and although anti-FIIa activity was in most carriers within the normal range, 7 cases (4 with the c.883G>A mutation and 3 with the c.881G>T mutation) had values below the normal range (<80 U/dL). Consequently, anti-FIIa activity was overall slightly lower in carriers than in controls (Table 1).

**Table 1:** Functional studies in carriers and non-carriers of the *SERPINC1* mutations

	Control (n = 30)	c.883G>A (n = 12)	c.881G>T (n = 5)^*^	c.880C>T (n = 2)	c.881G>A (n = 1)
**AT antigen levels (%)**	102.3 ± 9.8	97.4 ± 11.7	93.8 ± 6.4	91.5 ± 13.4	81.2
**(range)**	(82.6-121.5)	(79.1-116.0)	(87.3-101.6)	(81.8-101.5)	
		P = 0.1737	P = 0.0716	P = 0.1473	
**Anti-IIa + UFH**	105.7 ± 11.3	89.2 ± 17.5	77.0 ± 10.8	90.5 ± 9.2	88
**(range)**	(88-131)	(65-124)	(65-91)	(84-97)	
		P = 0.0008	P < 0.0001	P = 0.07	
**Anti-Xa + LMWH**	113.5 ± 10.3	106.5 ± 16.1	124.3 ± 20.0	117.0 ± 21.2	ND
**(range)**	(86.4-128.3)	(75.3-136.3)	(103.7-146.2)	(101.7-131.5)	
		P =0.10	P = 0.36	P = 0.66	
**Peak of thrombin**	258.0 ± 44.6	699.0 ± 509.4	513.6 ± 262.7	ND	ND
**(range)**	(173.1-408.3)	(224.0-1669.2)	(222.5-733.1)		
		P = 0.03	P = 0.23		
**Time to peak**	4.48 ± 0.60	6.19 ± 1.64	6.30 ± 1.10	ND	ND
**(range)**	(3.33-6.11)	(4.17-9.33)	(5.33-7.88)		
		P = 0.01	P = 0.0005		
**ETP**	1161.2 ± 188.0	3135.5 ± 2050.0	2324.2 ± 818.6	ND	ND
**(range)**	(768.4-1640.9)	(1141.1-7423.7)	(1568.0-3193.5)		
		P = 0.02	P = 0.12		

^*^The plasma sample from a carrier of c.881G>T was not available. Values (mean ± SD) represent percentages of the result obtained in a pool of 100 healthy controls. AT, antithrombin; UFH, unfractionated heparin; LMWH, low-molecular-weight heparin. ETP: Endogenous thrombin potential

ND: Not Determined.

**Figure 3:**
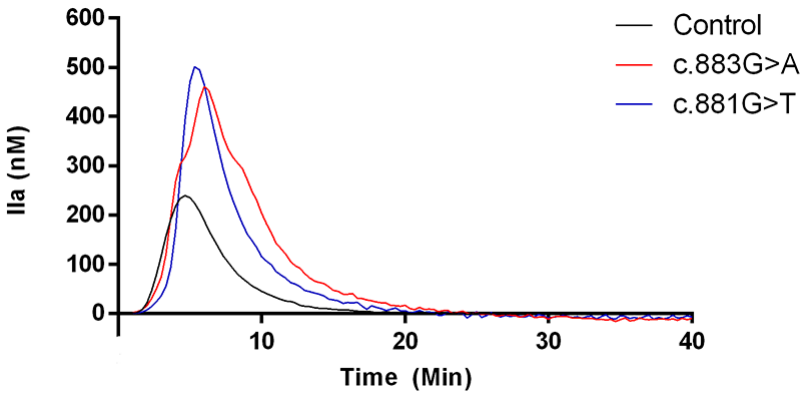
Representative thrombin generation of a control and two carriers of c.883G>A and c.881G>T mutations

**Figure 4:**
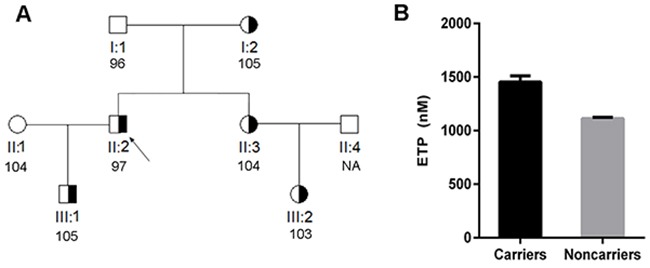
Anticoagulant capacity of carriers and non-carriers of the c. 880C>T mutation from a single family **(A)** Pedigree of the family. The proband is identified with an arrow. The anti-FIIa activity of each subject is also shown. **(B)** Endogenous thrombin potential (ETP) of carriers and non-carriers of c.880C>T from this family. ^*^ P < 0.001.

Thrombin generation assays revealed really interesting information. VTE patients that carried the highly recurrent mutations (c.883G>A p.Val295Met and c.881G>T p.Arg294Leu) had a 2.0~2.7-fold higher thrombin generation in response to tissue factor than healthy controls (Table 1 and Figure 3). In agreement with these results, thrombin generation assays performed in relatives of a carrier of the c.880C>T (p.Arg294Cys) mutation confirmed that carriers (N = 5) in this family had significantly higher endogenous thrombin potential (ETP) than non-carriers (N = 3), while anti-FIIa activity was similar in all subjects (Figure 4).

### Recombinant expression of *SERPINC1* variants

**Figure 5:**
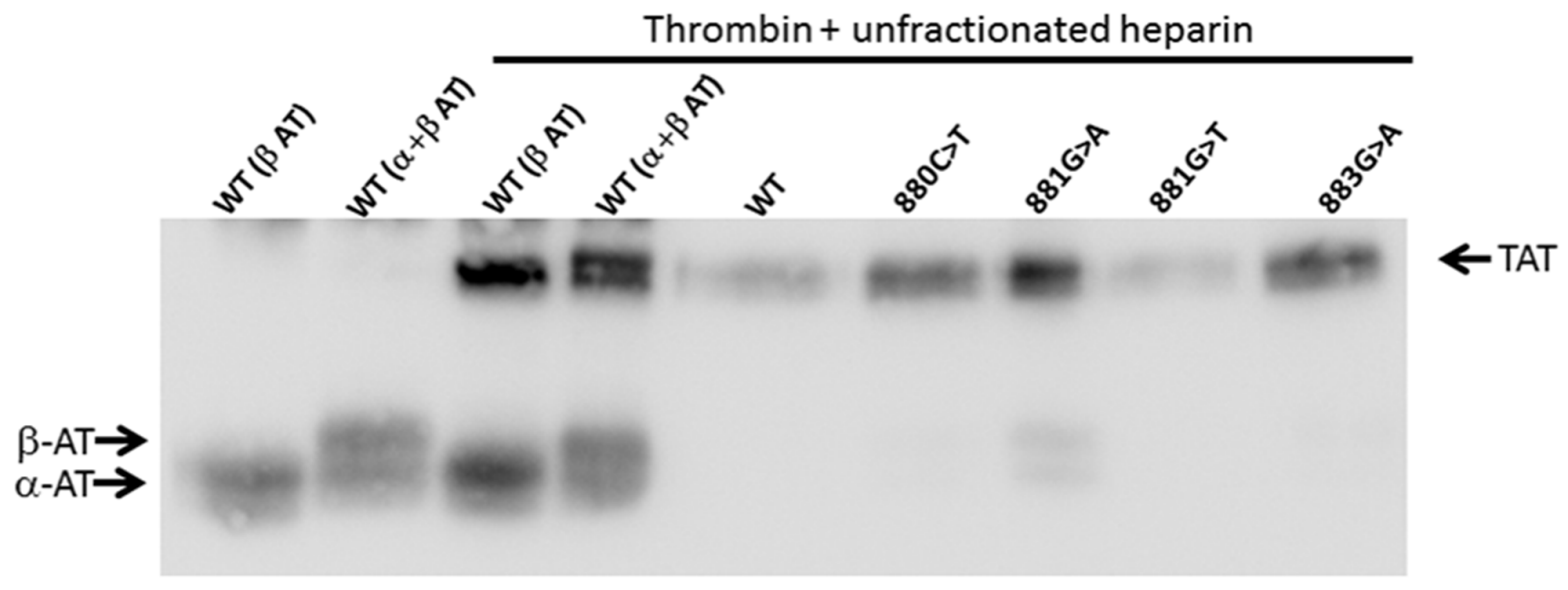
Formation of thrombin-antithrombin complexes by recombinant wild type and variant antithrombins Conditioned medium of HEK-EBNA cells transfected with plasmids carrying different *SERPINC1* mutations was incubated for 5 min with human thrombin and UFH. Antithrombin (AT) glycoforms (α and β) and thrombin-antithrombin complex (TAT) pointed by arrows were detected after SDS-PAGE by Western blot using a polyclonal antibody against human antithrombin.

To assess whether the p.Val295Met, p.Arg294Leu, p.Arg294His, and p.Arg294Cys *SERPINC1* mutations identified in our study affected the expression of the anti-thrombin protein transcript, recombinant expression of these four variants was evaluated in HEK cells expressing the Epstein Barr Nuclear Antigen 1 (HEK-EBNA). Results showed that the expression of the four recombinant proteins was comparable to that of wild type antithrombin. Moreover, all these recombinant molecules were able to form thrombin-antithrombin complexes in the presence of UFH (Figure 5).

## DISCUSSION

Despite modern methods of massive genetic screening, new prothrombotic mutations have been rarely identified in the last decade [32, 33]. As most of these studies have been restricted to the Caucasian population, screening of other populations may help to identify not only new genetic risk factors potentially affecting millions of people in societies where the rate of venous thrombosis is increasing, but also to describe new mechanisms underlying VTE.

There are evident ethnic differences between Asians and Caucasians in the genetic features of VTE. Three prothrombotic *SERPINC1* polymorphisms found in Caucasians, namely factor V Leiden, prothrombin G20210A, and antithrombin Cambridge II (p.Ala416Ser), are not found or rarely reported among Asians [20, 34, 35]. In contrast, two prothrombotic polymorphisms in *PROC* seem to be restricted to the Asian population [36, 37]. There are also significant differences concerning severe thrombophilia. The prevalence of antithrombin, protein C, and protein S deficiencies has been reported to be higher in the general population or in patients with VTE from China, Japan, and Thailand than in Caucasians [9]. In this study, we have identified a hotspot in *SERPINC1* that significantly and severely increases the risk of VTE in the Chinese population. According to our large case-control study, three close mutations, c.883G>A (p.Val295Met), c.881G>T (p.Arg294Leu), and c.880C>T (p.Arg294Cys) increase almost 20-fold the risk of VTE. Among them, c.880C>T (p.Arg294Cys) was once reported in a Japanese patient with VTE [23]. However, they are nearly absent in the Caucasian cohort including 160 antithrombin deficiency patients, 100 VTE patients and 100 healthy subjects.

More interesting than this association is, in our opinion, the fact that these mutations are not detected by current methods to diagnose antithrombin deficiency: antigenic assays and functional methods evaluating anti-FXa and anti-FIIa activities. In agreement with our results, the anticoagulant activity of the Japanese carrier of c.880C>T (p.Arg294Cys) mutation was also within the normal range.[23] In addition, the recombinant model supports that these mutations have negligible consequences on *SERPINC1* expression and anti-thrombin activity, at least under our experimental conditions. These data are consistent with recent studies indicating that other pathological mutations affecting *SERPINC1* are not detected by functional methods [26]. Interestingly, the thrombin generation assay revealed that these mutations significantly impair the anticoagulant capacity, leading to increased generation of thrombin, which explains the associated risk of thrombosis. It is now apparent that current functional methods, based on the inhibition of a single protease under specific conditions (with predetermined concentrations and incubation conditions for specific cofactors) fail in unmasking the deleterious consequences of some *SERPINC1* mutations. Indeed, the p.Val295Met mutation was determined to be ‘damaging’ and ‘probably damaging’ according to, respectively, SIFT and PolyPhen-2 predictions. Modification of these residues, located at the end of s1B, might impair the interaction of antithrombin with target proteases, particularly with thrombin; the resulting reduction in its anticoagulant capacity is not revealed by current functional methods but can be instead detected by thrombin generation assays. Actually, the closely located residues Tyr285 and Glu287 belong to an exosite that augments protease interaction mediated by allosteric activation of antithrombin by heparin [38]. It is hence possible that mutations in Arg294 or Val295 impair the interaction of native antithrombin with certain proteases, particularly thrombin. Further studies are required to verify this hypothesis.

All these data, together with recent evidence that highlights the limitations of current functional methods to detect pathological mutations in *SERPINC1* [23, 26], as well as the presence of *SERPINC1* mutations that only achieve pathogenic consequences under specific conditions [39], strongly suggest that defects on antithrombin as the underlying cause of VTE are actually underestimated. Antithrombin is critical in the physiopathology of the hemostatic system because of its highly efficient anticoagulant mechanism and the wide variety of procoagulant proteases that is able to inhibit. The risk of VTE is increased not only by severe, but also by mild antithrombin deficiency [40] or even by low borderline plasma levels of antithrombin [41, 42], which have been associated with a small but statistically significant increased risk of VTE. Our study supplies further evidence for the potential contribution of antithrombin polymorphisms to VTE, by stressing that mutations that apparently have minor or no functional effect may significantly increase its risk; moreover, current laboratory diagnostic tests are often insufficient to predict whether these mutations carry deleterious consequences. Therefore, development of new functional methods to diagnose antithrombin deficiency is warranted, and the thrombin generation assay may be an excellent platform in this regard. Additionally, the use of molecular diagnostic methods in cases where current functional methods yielded negative results should help clarify the real prevalence of antithrombin defects in VTE.

## MATERIALS AND METHODS

### Subjects

All the participants gave written informed consent to enter the study, which was approved by the institutional review boards of the Ethics Committee of the Union Hospital affiliated with Huazhong University of Science and Technology, or the Hospital Reina Sofia, University of Murcia, Spain. The demographic features, sample collection, and criteria for diagnosis of VTE have been previously described in detail [29, 30]. Briefly, blood samples were taken at least one month after the acute VTE event, and none of the subjects received anticoagulant therapy for at least two weeks prior to the time of admission. Objective diagnosis of VTE was mandatorily based on clinical manifestations, D-dimer levels, and the results of compression ultrasonography, ventilation/perfusion lung scan, or spiral computed tomography (CT). Patients with heparin treatment, chronic liver diseases, or nephrotic syndrome were excluded. Age- and sex-matched healthy volunteers, who had no documented personal or family history of thrombosis in first-degree relatives, were enrolled as controls.

Our study included 1,304 VTE individuals and 1,334 controls from China. The acquired risk factors for VTE, including smoking, malignancy, immobility, pregnancy or puerperium, oral contraceptives, hormone-replacement therapy, and lupus anticoagulant, were also documented in cases and controls.

Additionally, a cohort of Caucasian subjects was also enrolled in this study: 160 patients with antithrombin deficiency, 100 patients with VTE but no antithrombin deficiency, and 100 healthy controls.

### Genotyping and sequencing

Our first aim was to genotype in the whole case control study the *SERPINC1* c.883G>A (p. Val295Met) mutation identified in a previous study [29] in two out of 190 VTE patients with normal antithrombin activity (normal range: 80–120 U/dL). We therefore amplified exon 5 of *SERPINC1* using the following oligonucleotide primers: forward primer, 5’-TCATTCTGACACAGCCATT-3’; and reverse primer, 5’-CCTGACTTGTTGCTCCTTT-3’. Identification of the c.883G>A mutation was done by restriction analysis of the 649-bp PCR product with BstUI (New England Biolabs, Ipswich, MA, USA). Briefly, 10 μL PCR product was completely digested with 2 U of BstUI during 2 hours at 60°C. The digestion products were separated by electrophoresis in 2% agarose gels. Two fragments of 371 and 278-bp were generated by the wild type G allele, while the A allele, which disturbs the sequence recognized by BstUI, generated a single band of 649-bp (Figure 1). All cases with a potentially mutated allele were verified by direct sequencing of the PCR product.

Three common prothrombotic polymorphisms in the Chinese population, two affecting the gene encoding protein C (*PROC* c.565C>T, and c.574_576del) and one affecting the gene encoding thrombomodulin (*THBD* c.-151G>T) were also genotyped in patients and controls, as reported previously [30, 36, 37].

### Plasma antithrombin measurements

Plasma anti-FIIa activity was measured with the commercial reagent STA-STACHROM® AT III on the STA-R evolution automatic coagulation analyzer (Diagnostica Stago, Asnières, France), in the presence of unfractionated heparin (UFH) following the manufacturer’s instructions. Anti-FXa activity with low molecular weight heparin (LMWH) was measured using a chromogenic substrate method (Hyphen Biomed, Neuville-sur-Oise, France). Plasma from 100 healthy donors was pooled to generate the reference sample used to build the standard curve for the assays.

Immunochemical determination of antithrombin antigen levels was performed using a Human Antithrombin III ELISA Kit (AssayPro, St. Charles, MO, USA).

Functional and antigenic assays were done in plasma from 20 carriers of *SERPINC1* mutations affecting the hotspot identified in this study, as well as in 30 healthy controls.

### Thrombin generation assay

The thrombin generation assay was performed to evaluate the influence of the *SERPINC1* mutations c.883G>A and c.881G>T on blood coagulation with a calibrated automated thrombography system (Thrombinoscope BV, Maastricht, Netherlands), as previously described [29]. Briefly, in each well of a 96-well round-bottomed plate, 80 μL of plasma was mixed with 20 μL of PPP reagent (tissue factor 5 pM; phospholipids 4 μM). Coagulation was initiated by adding 100 mM calcium chloride (20 μL) in a custom BSA buffer containing 2.5 mM fluorogenic substrate. Thrombin generation was followed for 40 min at 37 °C.

### Recombinant expression of antithrombin variants

Recombinant expression of the four *SERPINC1* mutations identified in the present study was done in Human Embryonic Kidney cells expressing the Epstein Barr Nuclear Antigen 1 (HEK-EBNA). The *SERPINC1* mutations were generated into the *SERPINC1* cDNA (SERPINC1 open reading frame expression-ready clone; GeneCopoeia, Rockville, MD, USA) by site-directed mutagenesis using appropriate primers (Supplementary Table 1). The resulting plasmids were sequenced to confirm correct mutagenesis. HEK-EBNA cells were grown in DMEM with GlutaMAX-I medium (Invitrogen, Prat de Llobregat, Barcelona, Spain) supplemented with 5% fetal bovine serum (Sigma-Aldrich) to 60% confluence at 37 °C and 5% CO_2_ in a humidified incubator. Then, 200 μg/ml of wild-type or mutant plasmids were transfected for 30 min in OptiMEM with Lipofectamine LTX (Invitrogen), following the manufacturer’s recommendations. After 24 hours, cells were washed with PBS and exchanged into CD-CHO medium (Invitrogen) supplemented with 4 mM L-glutamine and 0.25 mg/ml Geneticin (Invitrogen). Cells were grown for 2 days and culture medium was collected. Cells were extensively washed with sterile PBS and then lysed with 50 μl of lysis buffer (10 mM Tris-HCl, 0.5 mM DTT, 0.035% SDS, 1 mM EGTA, 50 mM sodium fluoride, 50 μM sodium orthovanadate, 5 mM benzamidine, and 20 mM phenylmethylsulfonyl fluoride).

Antithrombin in lysates and conditioned media was evaluated by SDS-PAGE and Western blot. Briefly, after separation, proteins were transblotted onto a polyvinylidene difluoride membrane. Antithrombin was immunostained with a rabbit anti-human antithrombin polyclonal antibody (Sigma-Aldrich) followed by donkey anti-rabbit IgG–horseradish peroxidase conjugate (GE Healthcare, Madrid, Spain), and signal revealed with an ECL kit (Amersham Biosciences, Piscataway, NJ, USA). The anticoagulant activity of the variant secreted to the medium was assayed by evaluating the formation of thrombin-antithrombin complexes by SDS-PAGE and Western blot after incubation of medium with human thrombin and UFH for 5 minutes.

### Statistical analysis

Statistical analyses were performed with SPSS 13.0 for Windows (SPSS Inc, Chicago, IL, USA). Continuous variables were expressed as means ± standard deviation (SD), and discrete variables were expressed as counts (percentages). The unpaired Student’s t test or Mann-Whitney U test were performed to compare continuous variables, and the Pearson’s chi-square test or the Fisher exact test were used to compare allele and genotype distributions in patients and controls. Multivariate analysis was performed using multiple logistic regression and including all the significant covariates. The coefficients obtained from the logistic regression were expressed in terms of odds ratios (ORs) with 95% confidence intervals (CIs). All the results are expressed as 2-tailed values. Statistical significance level was set at P < 0.05.

## SUPPLEMENTARY MATERIALS FIGURES AND TABLES



## Abbreviations

VTE: venous thromboembolism
DVT: deep vein thrombosis
PE: pulmonary embolism
UFH: unfractionated heparin
LMWH: low molecular weight heparin
ETP: endogenous thrombin potential
