# Correction: Recurrent mutations in a *SERPINC1* hotspot associate with venous thrombosis without apparent antithrombin deficiency

**DOI:** 10.18632/oncotarget.24519

**Published:** 2018-02-16

**Authors:** Wei Zeng, Bei Hu, Liang Tang, Yan-Yan You, Mara Toderici, Maria Eugenia de la Morena-Barrio, Javier Corral, Yu Hu

**Affiliations:** ^1^ Institute of Hematology, Union Hospital, Tongji Medical College, Huazhong University of Science and Technology, Wuhan, China; ^2^ Department of Radiation and Medical Oncology, Zhongnan Hospital of Wuhan University, Wuhan, Hubei, China; ^3^ Department of Anesthesiology, Tongji Hospital, Tongji Medical College, Huazhong University of Science and Technology, Wuhan, China; ^4^ Centro Regional de Hemodonaciόn, Universidad de Murcia, IMIB, CIBERER, Murcia, Spain

**This article has been corrected:** the 1^st^ affiliation has been revised.

Original article: Oncotarget. 2017; 8:84417-84425. https://doi.org/10.18632/oncotarget.21365

