# Supplementary material for: Recurrent mutations in a SERPINC1 hotspot associate with venous thrombosis without apparent antithrombin deficiency
Source: Oncotarget. 2017 Sep 28;8(48):84417–25. doi: 10.18632/oncotarget.21365 (PMC5663607; doi:10.18632/oncotarget.21365)
Supplement: Supplementary file 1 [file oncotarget-08-84417-s001.pdf]

Recurrent mutations in a *SERPINC1* hotspot associate with venous thrombosis without apparent antithrombin deficiency

SUPPLEMENTARY MATERIALS

Supplementary Table 1: PCR primers and conditions used for constructing recombinant expression of antithrombin variants

Primer	Forward sequence (5′→3′)	Reverse sequence (5′→3′)	Annealing temp. (°C)	Product size (bp)
883G>A	**TTATCGGCGC*A*TGGCTGAAGGCACCCAGGTG**	**CCTTCAGCCA*T*GCGCCGATAACGGAACTTGC**	68	7218
881G>T	**CGTTATCGGC*T*CGTGGCTGAAGGCACCCAG**	**TTCAGCCACG*A*GCCGATAACGGAACTTGCCTT**	68	7218
881G>A	**CGTTATCGGC*A*CGTGGCTGAAGGCACCCAG**	**TTCAGCCACG*T*GCCGATAACGGAACTTGCCTT**	68	7218
880C>T	CCGTTATCGG***T***GCGTGGCTGAAGGCACCCA	CAGCCACGC***A***CCGATAACGGAACTTGCCTTC	68	7218

The underlined and italicized bases were used introduce the mutations.
